# Redesigning N-glycosylation sites in a GH3 β-xylosidase improves the enzymatic efficiency

**DOI:** 10.1186/s13068-019-1609-2

**Published:** 2019-11-14

**Authors:** Marcelo Ventura Rubio, César Rafael Fanchini Terrasan, Fabiano Jares Contesini, Mariane Paludetti Zubieta, Jaqueline Aline Gerhardt, Leandro Cristante Oliveira, Any Elisa de Souza Schmidt Gonçalves, Fausto Almeida, Bradley Joseph Smith, Gustavo Henrique Martins Ferreira de Souza, Artur Hermano Sampaio Dias, Munir Skaf, André Damasio

**Affiliations:** 1 Department of Biochemistry and Tissue Biology, Institute of Biology, University of Campinas (UNICAMP), Rua Monteiro Lobato, 255, Cidade Universitária Zeferino Vaz, Campinas, SP 13083-862 Brazil; 2 Department of Physics, Institute of Biosciences, Humanities and Exact Sciences, São Paulo State University (UNESP), São José do Rio Preto, SP 15054-000 Brazil; 3 Department of Medical Science, Faculty of Medicine, University of Campinas (UNICAMP), Campinas, SP 13083-862 Brazil; 4 Department of Biochemistry and Immunology, Ribeirão Preto Medical School, University of São Paulo (USP), Ribeirão Preto, SP 14049-900 Brazil; 5 Institute of Chemistry and Center for Computing in Engineering and Sciences, University of Campinas (UNICAMP), Campinas, SP 13084-862 Brazil

**Keywords:** β-Xylosidase, *Aspergillus nidulans*, N-glycosylation, Enzyme secretion, Glycomutants, CAZyme, Glycoside hydrolase family 3

## Abstract

**Background:**

β-Xylosidases are glycoside hydrolases (GHs) that cleave xylooligosaccharides and/or xylobiose into shorter oligosaccharides and xylose. *Aspergillus nidulans* is an established genetic model and good source of carbohydrate-active enzymes (CAZymes). Most fungal enzymes are N-glycosylated, which influences their secretion, stability, activity, signalization, and protease protection. A greater understanding of the N-glycosylation process would contribute to better address the current bottlenecks in obtaining high secretion yields of fungal proteins for industrial applications.

**Results:**

In this study, BxlB—a highly secreted GH3 β-xylosidase from *A. nidulans*, presenting high activity and several N-glycosylation sites—was selected for N-glycosylation engineering. Several glycomutants were designed to investigate the influence of *N*-glycans on BxlB secretion and function. The non-glycosylated mutant (BxlB^non-glyc^) showed similar levels of enzyme secretion and activity compared to the wild-type (BxlB^wt^), while a partially glycosylated mutant (BxlB^N1;5;7^) exhibited increased activity. Additionally, there was no enzyme secretion in the mutant in which the N-glycosylation context was changed by the introduction of four new N-glycosylation sites (BxlB^CC^), despite the high transcript levels. BxlB^wt^, BxlB^non-glyc^, and BxlB^N1;5;7^ formed similar secondary structures, though the mutants had lower melting temperatures compared to the wild type. Six additional glycomutants were designed based on BxlB^N1;5;7^, to better understand its increased activity. Among them, the two glycomutants which maintained only two N-glycosylation sites each (BxlB^N1;5^ and BxlB^N5;7^) showed improved catalytic efficiency, whereas the other four mutants’ catalytic efficiencies were reduced. The N-glycosylation site N5 is important for improved BxlB catalytic efficiency, but needs to be complemented by N1 and/or N7. Molecular dynamics simulations of BxlB^non-glyc^ and BxlB^N1;5^ reveals that the mobility pattern of structural elements in the vicinity of the catalytic pocket changes upon N1 and N5 N-glycosylation sites, enhancing substrate binding properties which may underlie the observed differences in catalytic efficiency between BxlB^non-glyc^ and BxlB^N1;5^.

**Conclusions:**

This study demonstrates the influence of N-glycosylation on *A. nidulans* BxlB production and function, reinforcing that protein glycoengineering is a promising tool for enhancing thermal stability, secretion, and enzymatic activity. Our report may also support biotechnological applications for N-glycosylation modification of other CAZymes.

## Background

Filamentous fungi possess several genes related to plant biomass degradation, which makes them important sources of CAZymes with high enzyme secretion capabilities [1]. However, enzyme yield is a significant bottleneck in economically feasible production of plant cell wall-degrading enzymes. Glycoside hydrolase family 3 (GH3) enzymes produced by *Aspergillus* spp.—such as β-xylosidase, β-glucosidase, α-l-arabinofuranosidase, and exo-1,3-1,4-β-glucanase—are important enzymes with diverse activities. GH3 enzymes with β-xylosidase activity (xylan 1,4-β-d-xylosidase, EC 3.2.1.37) perform hemicellulose degradation by hydrolyzing non-reducing ends of xylooligosaccharides and/or xylobiose, releasing xylose. These enzymes play a central role in plant biomass degradation, having applications in biofuel, paper, food, and animal feed industries [2].

Several post-translational modifications (PTMs) occur on microbial proteins, including those in bacteria and fungi. N-glycosylation is one of the most important PTMs—influencing protein secretion, stability, activity, signalization, and protease protection [3]. In addition, many proteins produced by filamentous fungi are N-glycosylated, including those generated and secreted by *Aspergillus nidulans* [4]. N-glycosylation is catalyzed by oligosaccharyltransferases in the lumen of the endoplasmic reticulum (ER), and involves the attachment of a glycan to an asparagine (*N*) residue; it is found in 70% of predicted N-glycosylation consensus sequences or sequons (N-X-S/T, where *X* is any amino acid except proline, and S/T is serine or threonine) [5].

The influence of *N*-glycans on CAZyme properties can be exemplified with several proteins. For example, a thermophilic GH10 xylanase from *Aspergillus fumigatus* expressed in *Pichia pastoris* has improved activity and thermal stability in the N-glycosylated form, in comparison with the non-glycosylated form [6]. In another example, the position of N-linked glycans can positively or negatively influence the processivity of a GH6 cellobiohydrolase from *Penicillium verruculosum* [7]. Moreover, engineered N-glycosylation sites were shown to improve the thermal stability of cutinase C from *Aspergillus oryzae* by inhibiting thermal aggregation [8].

*Aspergillus nidulans* is a model organism for studying the secretion of recombinant enzymes [9–11]. However, studies investigating the role of N-glycosylation in enzyme secretion by filamentous fungi are scarce [12–15]. A recent study used N-glycoproteomics and N-glycomics to determine N-glycosylation patterns of proteins secreted from *A. nidulans* grown in glucose, xylan, and NaOH-pretreated sugarcane bagasse. More than 50% of the 265 identified N-glycoproteins were classified as CAZymes [4]. Among them, some industrially relevant enzymes were highly secreted, making them relevant targets for investigating the influence of N-glycosylation on enzymatic properties and secretion. Here, a GH3 (BxlB^wt^) secreted by *A. nidulans* A773 with high activity toward *ρ*-nitrophenyl-β-d-xylopyranoside (ρNP-X), was selected as a model for N-glycosylation engineering. BxlB^wt^ harbors seven putative N-glycosylation sites validated by LC–MS/MS. Site-directed mutagenesis was used to produce BxlB glycomutants exhibiting different N-glycosylation profiles, which were then used to investigate whether the absence or addition of specific N-glycosylation sites would influence enzyme production, secretion, kinetics, and stability. We observed two N-glycosylation sites that were important for BxlB catalytic efficiency. In addition, we observed that BxlB synthesis may be completely compromised by changing the N-glycosylation context.

## Results

### BxlB^wt^ has seven predicted N-glycosylation sites

BxlB^wt^ is one of the five most secreted enzymes by *A. nidulans* when cultivated on different polymeric substrates, and is the most secreted hemicellulase during cultivation on beechwood xylan [4]. BxlB^wt^ is a highly N-glycosylated enzyme belonging to the GH3 family, presenting seven predicted N-glycosylation sites (NetNGlyc 1.0 Server)—all of them were validated by mass spectrometry: N63, N340, N408, N458, N419, N621 and N760.

**Fig. 1:**
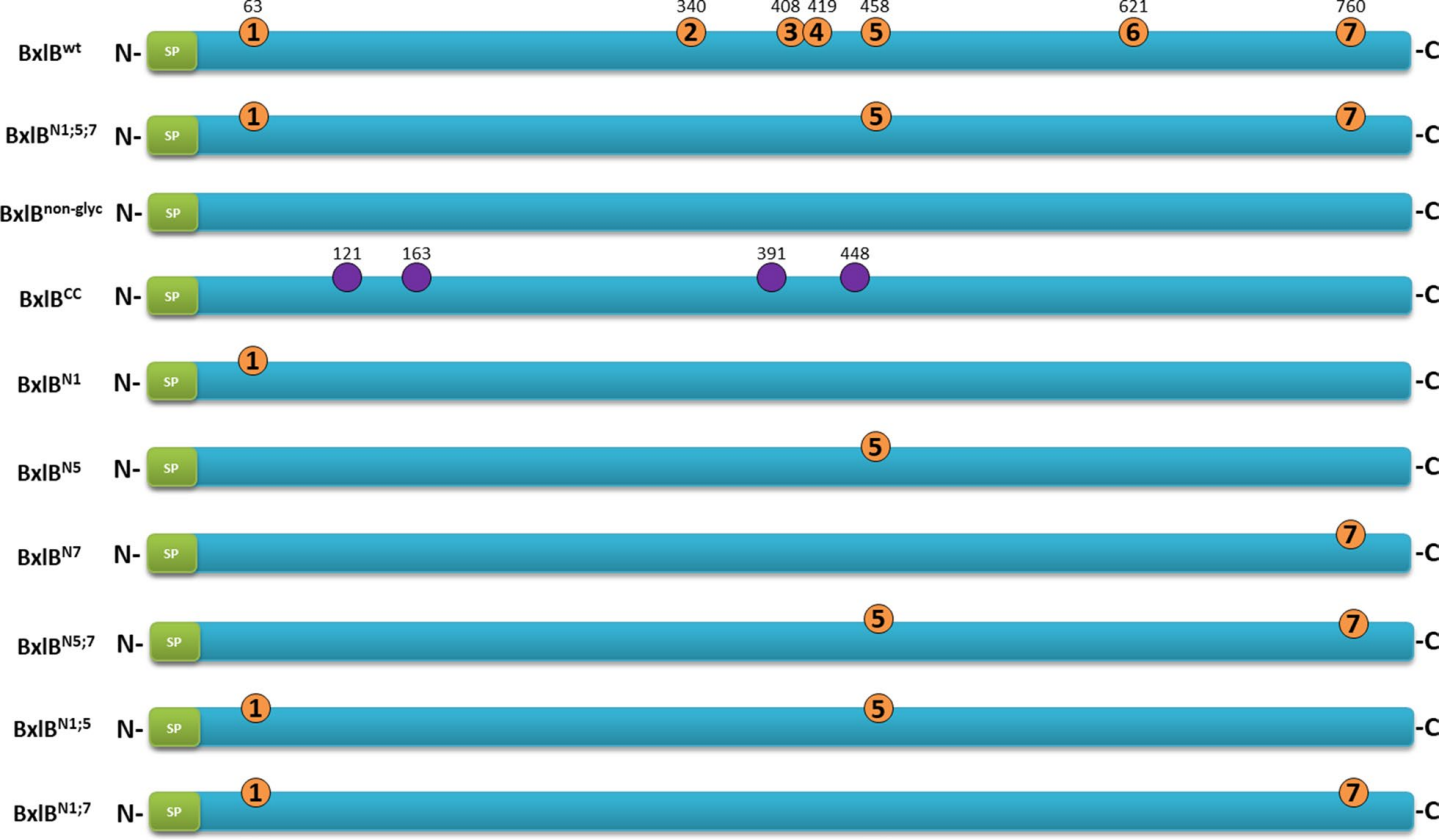
Overview of BxlB glycomutants. BxlB^wt^ N-glycosylation sites were predicted by the NetNGlyc server, and all of these sites were validated by LC–MS/MS (orange circles). Three glycomutants were synthesized: BxlB^N1;5;7^, N-to-Q mutation of four validated N-glycosylated sites; BxlB^non-glyc^, N-to-Q mutation of the seven predicted N-glycosylation sites; BxlB^CC^, addition of four new sites using BxlB^non-glyc^ as a template, to change the N-glycosylation context (purple circles), N121 (A123T), Q166 N, Q391 N, and N448 (L450T). Six additional mutants were designed by using the BxlB^non-glyc^ as a template maintaining individual N-glycosylation sites (BxlB^N1^, BxlB^N5^, BxlB^N7^) or combining two sites (BxlB^N1;5^, BxlB^N5;7^, BxlB^N1;7^). SP: signal peptide, N: amino-terminus, C: carboxy-terminus

N-glycosylation sequons were mutated by replacing asparagine (N) with glutamine (Q) and three BxlB glycomutants were designed: BxlB^non-glyc^, a non-glycosylated variant in which all N-glycosylation sites were mutated; BxlB^N1;5;7^, a partially glycosylated variant in which N340, N408, N419, and N621 were mutated; and BxlB^CC^, a variant in which four new N-glycosylation sites were added using BxlB^non-glyc^ as template. The design of the new sites was based on the homology with 33 β-xylosidases sequences from aspergilli (Additional file 1: Figure S1). In addition, the accessible surface area (ASA) for each new N-glycosylation site was calculated (Fig. 1 and Additional file 1: Table S1). The design of BxlB^CC^ enabled the explicit verification of the importance of N-glycosylation position for β-xylosidase production, secretion, and function.

### Production of BxlB glycomutants in *A. nidulans*

**Fig. 2:**
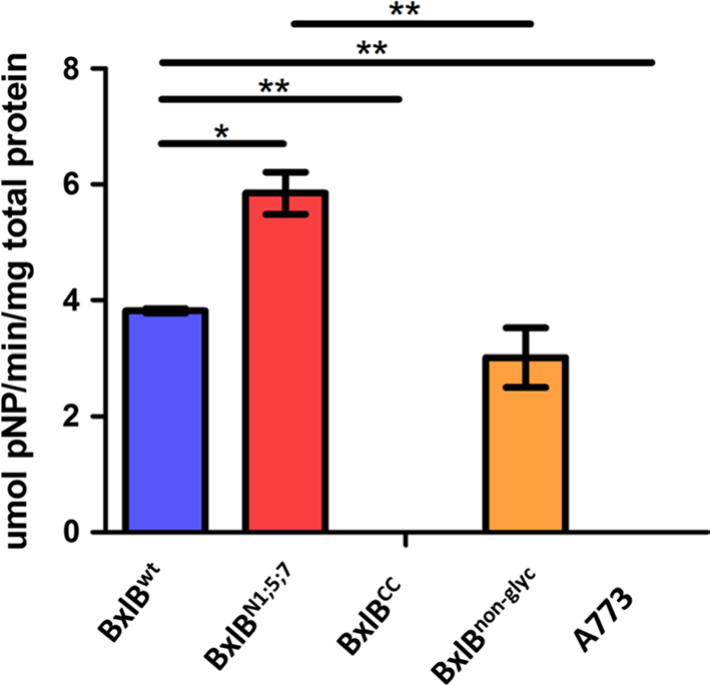
Analysis of BxlB glycomutant secretion by homologous expression in *A. nidulans*. The secretion of BxlB glycomutants was quantified by the ρNP-X assay. The reaction was carried out for 15 min at 50 °C and pH 5.0. *ANOVA with Bonferroni post hoc test, *p *< 0.05. * *p* ≤ 0.05; ** *p* ≤ 0.002; *** *p* ≤ 0.0002 and **** *p* ≤ 0.0001

All *bxlB* gene variants were cloned into the pEXPYR vector controlled by the *glaA* promoter and the glucoamylase signal peptide (SP) from *A. niger*, and then transformed into the *A. nidulans* A773 parental strain, before being cultivated with the inducer maltose [11]. Analyses of crude supernatants revealed that the non-glycosylated mutant (BxlB^non-glyc^) presented similar levels of activity to the wild type (Fig. 2), while the BxlB^N1;5;7^ mutant showed 1.5-fold higher enzymatic activity. Although BxlB^CC^ activity could not be detected, the identification of some peptides was verified by mass spectrometry (Additional file 1: Table S2).

In addition, very low BxlB^CC^ intracellular activity was detected (similar to the parental strain) (Additional file 1: Figure S2), and intermediate activity levels were found for BxlB^wt^ and BxlB^non-glyc^. BxlB^N1;5;7^, in turn, presented fourfold higher intracellular activity in relation to BxlB^wt^. Real-time PCR revealed that all BxlB glycomutants were properly transcribed, and no direct relationship between gene expression and enzyme production could be observed (Additional file 1: Figure S3).

### Understanding the BxlB^N1;5;7^ higher catalytic efficiency

Enzymatic activity was measured at pH 5.0 and 60 °C for all the BxlB glycomutants and the wild type. K_m_ values for BxlB^wt^, BxlB^N1;5;7^, and BxlB^non-glyc^ were 2.06, 2.49, and 2.36 mM, respectively, while corresponding V_max_ values were 9.55, 17.28, and 10.12 U/mg prot. Thus, the BxlB^N1;5;7^ glycomutant showed 50% higher catalytic efficiency as compared to the wild type, while BxlB^non-glyc^ catalytic efficiency was reduced by 7% (Table 1).

**Table 1:** Overview of kinetic parameters of BxlB glycomutants

	K_m_ (mM)	V_max_ (U/mg)	k_cat_ (1/s)	k_cat_/K_m_	k_cat_/K_m_ (%)^a^
BxlB^wt^	2.06 ± 0.13	9.55 ± 0.18	13.08	6.34	100
BxlB^N1;5;7^	2.49 ± 0.43	17.28 ± 1.07	23.67	9.51	150
BxlB^non-glyc^	2.36 ± 0.11	10.12 ± 0.14	13.86	5.87	93
BxlB^N1^	2.53 ± 0.23	9.49 ± 0.28	13.01	5.13	81
BxlB^N5^	2.71 ± 0.21	6.08 ± 016	3.71	0.61	10
BxlB^N7^	3.19 ± 0.22	3.42 ± 0.09	4.37	1.27	20
BxlB^N5;7^	2.81 ± 0.11	20.47 ± 0.28	28.04	9.94	157
BxlB^N1;5^	2.91 ± 0.18	21.95 ± 0.51	30.07	10.33	163
BxlB^N1;7^	9.92 ± 1.05	4.12 ± 0.26	5.64	0.57	9

^a^Relative to BxlB^wt^

To better understand the improvement of the BxlB^N1;5;7^ catalytic efficiency, six additional glycomutants were designed by maintaining the following N-glycosylation sites: N1 (BxlB^N1^), N5 (BxlB^N5^), N7 (BxlB^N7^), both N5 and N7 (BxlB^N5;7^), both N1 and N5 (BxlB^N1;5^), and both N1 and N7 (BxlB^N1;7^) (Fig. 1). The genes encoding these proteins were also cloned into the pEXPYR vector and transformed into *A. nidulans* A773.

Analysis of kinetic parameters showed that, in comparison to the wild type, BxlB^N5^, BxlB^N7^ and BxlB^N1;7^ catalytic efficiencies were drastically reduced, and BxlB^N1^ catalytic efficiency was partially reduced. BxlB^N1;5^ and BxlB^N5;7^ displayed the highest catalytic efficiencies with increases of 63% and 57%, respectively (Table 1).

**Fig. 3:**
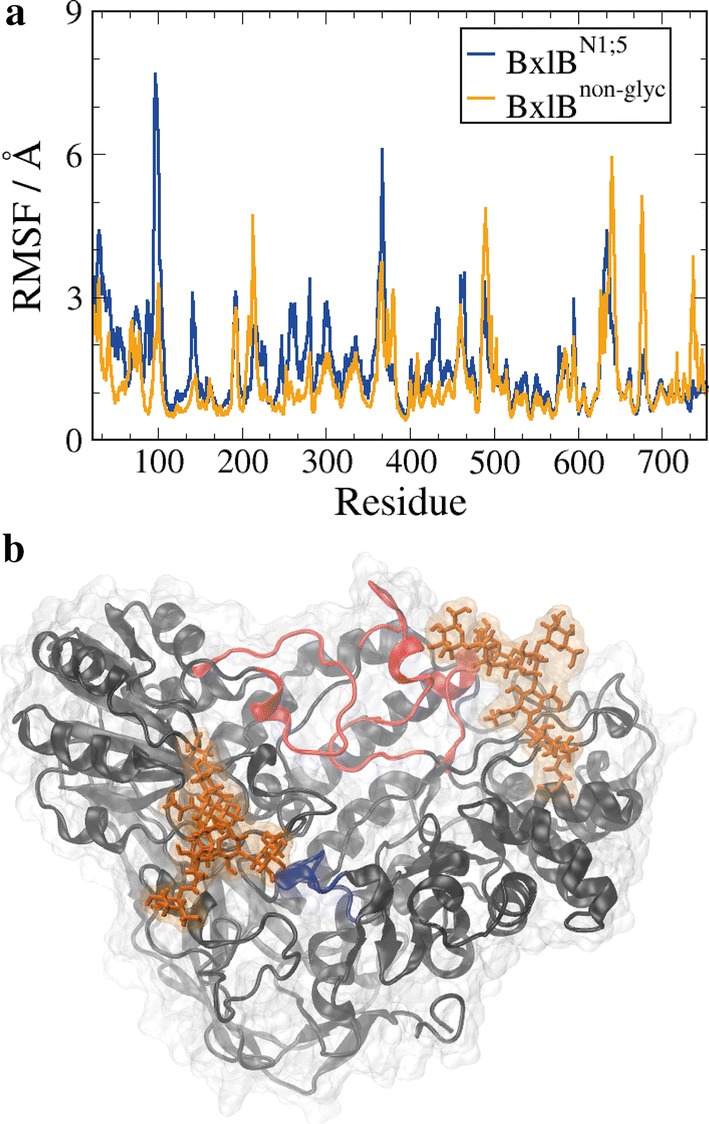
Flexibility patterns of BxlB^N1;5^ and BxlB^non-glyc^. **a** RMSF for BxlB^N1;5^ and BxlB^non-glyc^ residues. **b** Structural segments surrounding the catalytic which gain (loose) mobility upon glycosylation are shown in red (blue). The glycan structures are shown in orange in their respective N-glycosylation sites

To investigate how the N-glycosylation might affect the overall flexibility of the enzyme, we performed molecular dynamics (MD) atomistic simulations of both BxlB^N1;5^ and BxlB^non-glyc^ and computed the RMSF for each residue relative to the average structures (Fig. 3a). The presence of glycans at N1 and N5 increases the overall flexibility of the protein, especially in major segments that surround the catalytic site, but promotes stabilization of some structural elements of the enzyme (Fig. 3b).

### The BxlB^N1;5^ catalytic site is more stable than in BxlB^non-glyc^

**Fig. 4:**
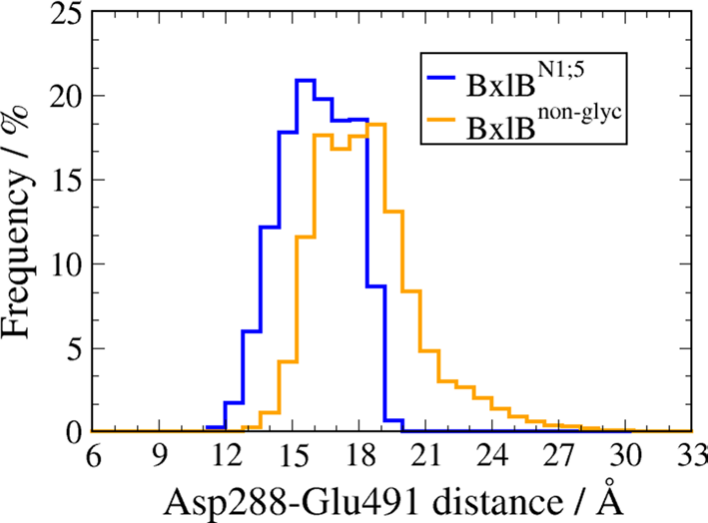
Distribution of distances between the α-carbons of the catalytic residues Asp288 and Glu491 along the last 350 ns of each simulation. The displacement toward lower distances upon N1 and N5 glycosylation is clear

The stability of the catalytic site in each system was examined by computing the distances between the α-carbons of the two catalytic residues—Asp288 and Glu491—along the molecular trajectories of both simulations. The average distances were 16.1 ± 1.6 Å and 18.4 ± 2.4 Å for the BxlB^N1;5^ and BxlB^non-glyc^ models, respectively, with a slightly narrower distribution for BxlB^N1;5^ (Fig. 4). The average distance between the corresponding Asp/Glu residues in other GH3 members, as measured from the crystal structures in PDB: 1IEX [16], 2X41 [17], 3ZYZ [18], 4I3G [19], 5AE6 [to be published], and 6Q7I [20]), is approximately 13 Å.

The N-glycosylation at N1 and N5 also contributes to enhance substrate binding, as the xylobiose ligand showed higher residence times inside the catalytic pocket of BxlB^N1;5^ than in BxlB^non-glyc^. In three out of four auxiliary MD runs, xylobiose remained bound to the catalytic pocket at least twice as long in BxlB^N1;5^ (Additional file 1: Figure S4). This is attributed to the glycan chains that surround the catalytic pocket and hinder the release of the substrate.

### N1 and N5 glycosylation affects the pattern of large-scale motions of the enzyme

**Fig. 5:**
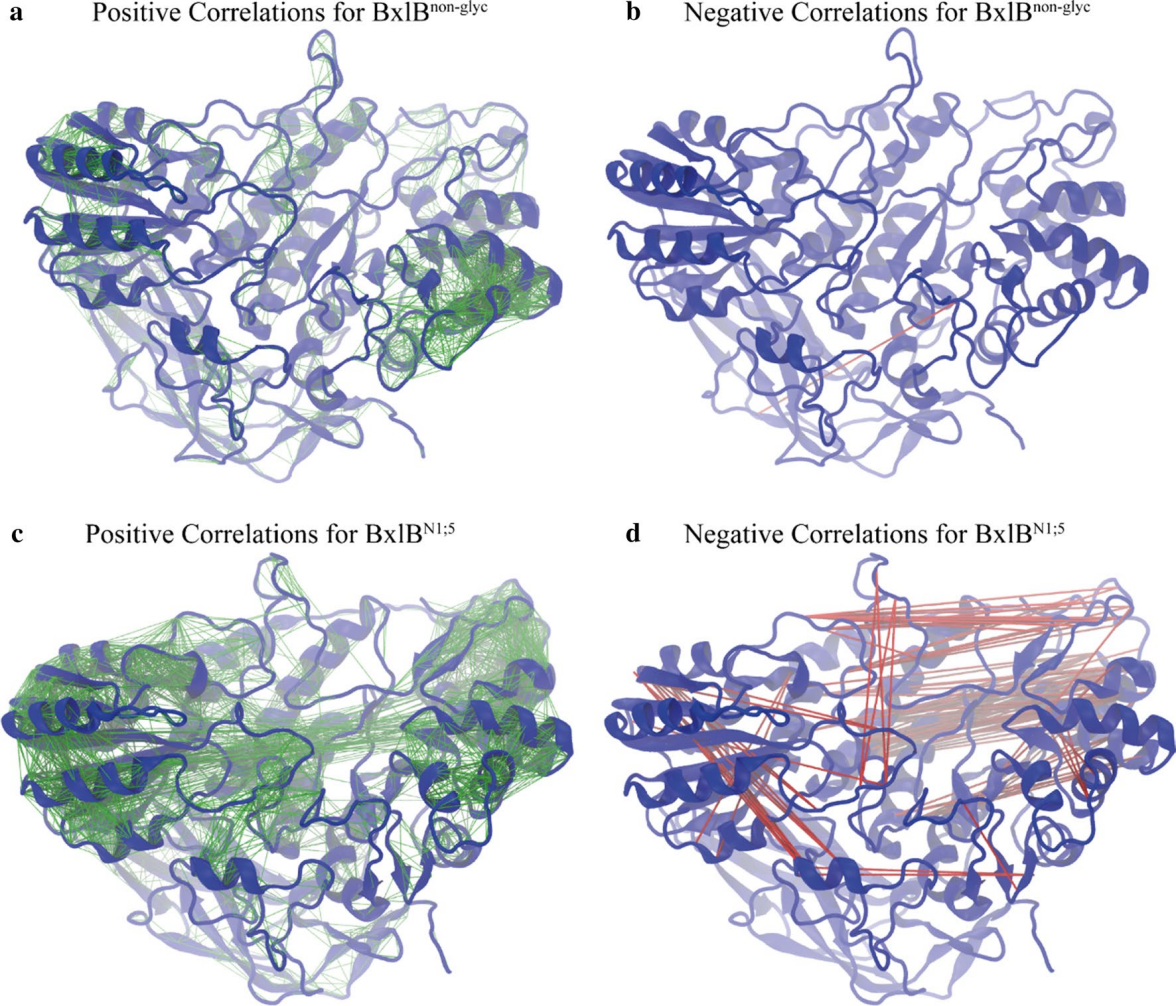
Large amplitude correlated motions in BxlB. Results are shown for pairwise correlation coefficients larger than 0.65. Green and red lines connect residues that exhibit positively and negatively correlated motions, respectively. **a** Positive correlations for BxlB^non-glyc^. **b** Negative correlations for BxlB^non-glyc^ (almost completely absent). **c** Positive correlations for BxlB^N1;5^. **d** Negative correlations for BxlB^N1;5^

The large-amplitude, low-frequency collective motions for both BxlB^N1;5^ and BxlB^non-glyc^ were investigated via principal components analysis of the α-carbons Pearson cross-correlations using the MD trajectories [21]. Positive correlations denote pairs of residues that move synchronously along the same direction, while negative correlations indicate pairs of residues that move synchronously along opposite directions. If two residues move independently, then the cross-correlation between them is null. BxlB^N1;5^ shows a very distinct pattern of collective motions when compared to BxlB^non-glyc^. The glycosylation at position N1 and N5 promotes a significant increase in the overall amount of positive correlations within the structural elements of the enzyme, but also gives rise to negative correlations between spatially distant parts of the structure, which were absent in BxlB^non-glyc^ (Fig. 5).

### Removal of N-glycosylation sites decreased BxlB thermal stability

Circular dichroism (CD) of the purified enzymes showed BxlB^wt^ and glycomutants to have similar secondary structure compositions, corresponding to 64% α-helix and 13% β-strand (Additional file 1: Figure S5) [22]. Nevertheless, BxlB glycomutant melting temperature range was around 8 °C, decreased from 63.8 °C for BxlB^wt^ to 61.6 °C for BxlB^N1;5;7^, and 59.5 °C for BxlB^non-glyc^. Six additional BxlB glycomutants also showed lower melting temperatures than the BxlB^wt^, from 61.1 °C for BxlB^N1;5^ until 55.1 °C for BxlB^N5;7^ (Additional file 1: Table S3).

### BxlB glycomutants showed lower secretion

**Fig. 6:**
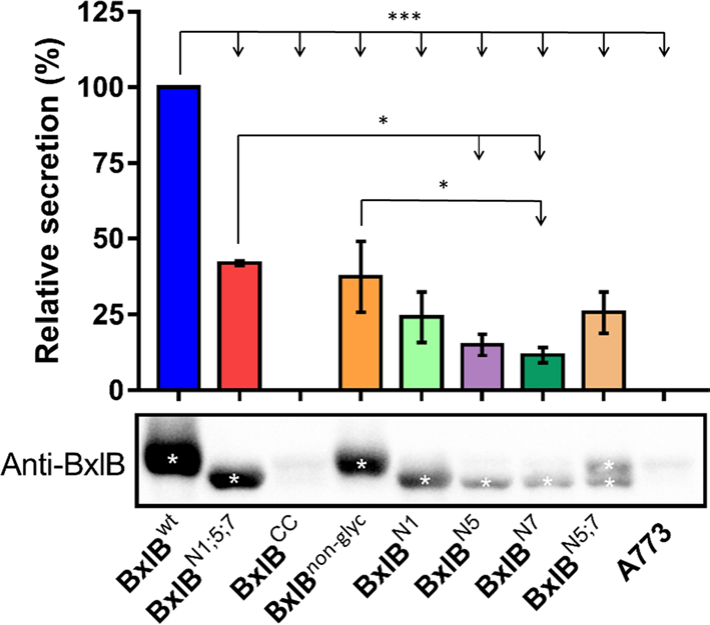
Secretion yield evaluated by western blot. WBs were performed in triplicate using BxlB glycomutant crude extracts (60 µg) and polyclonal BxlB antibody. The relative protein amount corresponding to the BxlB band was estimated using BxlB^wt^ as a reference. Asterisks in WB indicate the quantified band. A773: *A. nidulans* parental strain. *ANOVA with Tukey post hoc test, *p *< 0.05. * *p* ≤ 0.05; ** *p* ≤ 0.002; and *** *p* ≤ 0.0002

BxlB secretion in *A. nidulans* was evaluated by Western blot (WB) using a polyclonal BxlB antibody. All the glycomutants were secreted at lower levels than that in the wild type (Fig. 6). *In silico* analyses indicated that N-glycosylation assisted BxlB^wt^ folding by encouraging a more specific route with a lower free energy barrier compared to the non-glycosylated enzyme. The curve of specific heat capacity at constant volume—C_v_ (Additional file 1: Figure S6) showed that, for the wild type, there was a higher peak and a wider width that can be understood as a less cooperative protein folding. Following the wild-type analysis in particular, the free energy profile (Additional file 1: Figure S7) indicated a small shoulder in the curve around Q = 0.7, meaning that no intermediate states were being populated in the transition state, but that perturbations along the folding process may occur, similar to what happens for the other cases. Complementing both results, the *ϕ*-*value* analyses (Additional file 1: Figure S8) show for all cases that residues from the N-terminal portion (1 to 400) were involved in interactions during the transition state (TS), while the C-terminal portion (from 400 to the end of the chain) interactions for the wild type in the TS were insignificant, indicating a more specific route where the N-terminal portion was formed earlier. The reason is the limitation imposed upon the residue angles due to the N-glycosylation that restricts the conformational space, entropically favoring the process. Analyzing the other cases, it is possible to see an increase of residues from the C-terminal portion involved in contacts in the TS proportional to the removal of N-glycosylation. Thus, during folding, the protein is conducted to intermediate states resulting in traps by the competition of contacts from both sides, increasing the free energy profiles and providing an explanation for the C_v_ analysis that suggests a more cooperative process (narrowest barrier).

*In silico* data revealed the fundamental role of *N*-glycans during the protein folding and highlighted how N-glycosylation engineering can be a challenge. The evaluation was mainly focused on BxlB^wt^, BxlB^non-glyc^, BxlB^N1;5;7^, BxlB^N5;7^, and BxlB^N1^ forms. The constant volume specific heat analysis (Additional file 1: Figure S6) showed a higher thermal stability for BxlB^wt^ and BxlB^N1;5;7^ when compared to BxlB^non-glyc^, BxlB^N5;7^ and BxlB^N1^, corroborating to the results obtained experimentally (Additional file 1: Table S3). The inset in Additional file 1: Figure S6 shows the superimposition of the curves by critical temperature (T_c_) normalization. The width of the distribution indicates a slightly less cooperative folding process for BxlB^wt^, which in this case is favorable as previously presented. This result is complemented by the analysis of the free energy profiles (Additional file 1: Figure S7) and *ϕ*-*values* (Additional file 1: Figure S8).

BxlB^wt^ presents a lower free energy barrier in the transition state (*3.4 kT*) when compared to BxlB^non-glyc^ (*3.8 kT*), correlating to faster folding according to the Arrhenius equation. Even so, the BxlB^non-glyc^ free energy profile presents an intermediate state due a less harmonious folding process by the removal of restraints (higher conformational space) imposed by the glycans (Additional file 1: Figure S7). BxlB^wt^ has more specific folding, first forming C-terminal, for then finalize the process. Furthermore, the N-glycosylations in BxlB^wt^ seem to stabilize the folded and unfolded states. Since simulations are in reduced units the results are qualitative.

### Monitoring BxlB^CC^ secretion

The absence of detectable BxlB^CC^ secretion cannot be attributed to transcriptional impairment, as previously shown. Moreover, real-time PCR showed a non-significant difference in the transcription of *bipA* (ER chaperone) and *pdiA* (protein disulfide isomerase), genes normally associated with ER stress (data not shown) [23–25].

To further investigate the low secretion of BxlB^CC^, we asked if the enzyme was trapped inside the cell by WB analysis. Again, BxlB^CC^ was detected at a very weak intensity (Additional file 1: Figure S9). Furthermore, BxlB^wt^ and BxlB^CC^ were detected via immunohistochemistry. The polyclonal BxlB^wt^ antibody was identified with a red fluorescence antibody by confocal microscopy (Additional file 1: Figure S10 and Additional files 2, 3, 4, 5: Videos S1–4). Strong BxlB^wt^ signals were detected in *A. nidulans* hyphae, especially in the tips, which also had a very weak BxlB^CC^ signal, corroborating the WB.

## Discussion

### N-glycosylation enhances BxlB folding

The *BxlB*^wt^ gene from *A. nidulans* encoding β-xylosidase was selected from a set of 265 N-glycoproteins. The high levels of β-xylosidase secretion found when *A. nidulans* was cultivated on beechwood xylan provided evidence that this enzyme was highly important for biomass degradation [4]. β-Xylosidases have been detected in various prokaryotes and eukaryotes, but, according to the mycoCLAP database [26, 27], only 11 eukaryotic GH3 β-xylosidases have been characterized. Among them, four β-xylosidases have been expressed in hosts such as *Aspergillus* spp.*, Pichia pastoris*, and *Saccharomyces cerevisiae* (Additional file 1: Table S5). Here, a homologous expression system was used to prevent concerns related to accurate gene expression and protein production. In addition to the high secretion levels in the presence of lignocellulosic substrates, BxlB^wt^ can be considered a good model for studies regarding the influence of N-glycosylation on enzyme secretion and functional parameters, due to its considerable levels of N-glycosylation and enzymatic activity.

As observed in the free energy profiles (Additional file 1: Figure S7), the wild-type protein lacks an intermediate state after the transition state peak, thus showing a more specific and favorable folding process. This result is complemented by the *ϕ*-*values* analysis (Additional file 1: Figure S8) that indicates the formation of the N-terminal side of the protein preceding the folding of the C-terminal portion. The presence of an intermediate state after the transition state peak in the non-glycosylated protein’s free energy profile (red line in Additional file 1: Figure S7) indicates difficulties in achieving the folded state. The *ϕ*-*value* analysis (Additional file 1: Figure S8) also indicates simultaneous folding of the N- and C-terminal portions, allowing for a less specific folding process that traps the protein in an intermediate state. The non-glycosylated state enables a higher number of accessible conformations, suggesting that for some mutations folding may not occur. *N*-glycans play an important role by interacting with polypeptide chains, favoring promising folding pathways and the stabilization of the native and unfolded states. The secretion of glycoproteins is generally facilitated by their higher stability and the presence of calnexin/calreticulin quality control [28]. Furthermore, low thermodynamic stability is known to decrease protein export efficiency—i.e., very unstable proteins are poorly exported [29]. The influence of N-glycan composition on protein folding was also studied in the human immune cell receptor adhesion domain (CD2ad), and the presence of complete *N*-glycans was reported to provide a fourfold acceleration in folding, in addition to stabilizing the protein structure [30].

### *N*-glycans at positions 63, 458 and 760 are important for enzyme activity

BxlB^wt^, BxlB^N1;5;7^, BxlB^CC^ and BxlB^non-glyc^ secretion levels were evaluated after their homologous expression in *A. nidulans*. Activity assays of the extracellular enzymes revealed that complete deglycosylation did not affect BxlB^non-glyc^ activity. On the other hand, the improvement in BxlB^N1;5;7^ enzymatic activity compared to the wild type denotes that the N-glycosylation sites at N63, N458, and N760 are important for protein folding and export. Moreover, BxlB^CC^ activity and secretion were not detected in the extracellular fraction, indicating an impairment in either gene expression, protein production, or secretion. Real-time PCR showed high transcription levels for wild type, BxlB^N1;5;7^, BxlB^CC^, and BxlB^non-glyc^, suggesting that transcription is not a bottleneck for BxlB^CC^ production. There are few studies dealing with the influence of *N*-glycans on protein secretion, and variable results have been found. For example, changing two N-glycosylation sites (N14Q and N48Q) of a heterologous lipase expressed in *P. pastoris* did not affect its secretion, while the N60Q mutation completely abolished secretion [12]. Hence, it is possible that *N*-glycans attached to specific positions in the protein are be essential to folding kinetics and to the secretion pathway quality control.

### BxlB^wt^ deglycosylation reduces thermal stability

Secondary structure analyses of BxlB^wt^ and glycomutants revealed that all recombinant enzymes shared a classic profile, exhibiting α-helices as the predominant secondary structure (Additional file 1: Figure S5) [31]. Similarities in CD data were confirmed by deconvolution analysis, which found the same α-helix and β-strand rates (Additional file 1: Table S3). The deconvolution data were highly similar to what was found in *A. niger* β-xylosidase composed of 41% α-helix and 16% β-sheet; however, there was not as much similarity to the *T. reesei* β-xylosidase which presented 23% α-helix and 27% β-sheet composition [32, 33].

Generally, glycoproteins have higher stability than their partially glycosylated or non-glycosylated counterparts, despite the absence of structural links associated with N-glycosylation [34]. This was also observed in our study where all BxlB mutants were less stable when compared with the wild type (Additional file 1: Table S3). Recently, thermal stability was evaluated in *T. reesei* cellobiohydrolase (*Tr*Cel7A) by differential scanning calorimetry, with lower stability reported for all 15 N-glycosylation mutants [35]. Interactions between N-glycan sugars and amino acid residues often stabilize the protein structure; consequently, the glycosylated forms of a protein are more stable than the non-glycosylated counterpart [34, 36].

### Glycosylation at N63, N458, and N760 enhances BxlB kinetic parameters

The optimal activity conditions for all BxlB glycomutants were pH 5.0 and 60 °C using ρNP-X as substrate. The same enzyme expressed in *P. pastoris* showed optimal activity at pH 4.4 and 48 °C using rye arabinoxylan and xylohexaose as substrates [37]. This difference in reaction conditions due to N-glycosylation was also reported for β-glucosidase from *A. terreus* expressed in *P. pastoris* and *T. reesei* [13]. Kinetic parameters showed that the removal of any N-glycosylation site decreased BxlB^wt^ affinity for ρNP-X, indicating that *N*-glycans may facilitate substrate recognition or affect catalytic site flexibility (*Km* values in Table 1). Structural dynamics can influence positions and flexibility of catalytic residues, impacting kinetic parameters [38]. Wei et al. showed that removing some N-glycosylation sites decreased the specific activity of *A. terreus* β-glucosidase produced in filamentous fungi or yeast [13]. In accordance with this observation, we demonstrated that the removal of four N-glycosylation sites to form BxlB^N1;5;7^ increased V_max_ and K_m_, suggesting important changes to structural dynamics.

### N5 glycosylation site is essential to BxlB catalytic efficiency

Six additional mutants were designed to further understand the influence of each N-glycosylation site on folding, secretion, and BxlB^wt^ functional parameters. BxlB^N1^ secretion and catalytic efficiency decreased in comparison to BxlB^non-glyc^. Similarly, BxlB^N1;5;7^ secretion and catalytic efficiency decreased in comparison to BxlB^N5;7^. These data suggest that the N1 site has a slight negative influence on BxlB^wt^ kinetic parameters and secretion levels. However, for the other glycomutants that contained a single N-glycosylation site, maintaining N5 or N7 sites, showed a drastic negative influence on enzyme secretion and function. These alterations in secretion and enzyme activity were probably impacted by the N-glycan position in the 3D structure, as well as protein dynamics and stability [38, 39].

MD analysis of the catalytic site suggests that the N-glycosylation at positions N1 and N5 creates a net interaction that stabilizes the relative position of the catalytic residues, even though these two residues are not in the segments of the protein that had their flexibility mostly affected by the presence of *N*-glycans. That is, the suggested stabilization of the general acid/base catalytic pair may stem from the confluence of multiple long-range interactions, in which the *N*-glycans play a major role.

The highest activities were observed in the presence of N5 along with either N1 or N7, indicating that N5 is essential for BxlB dynamics, but is structurally stabilized by N1 or N7. As in the case of the essential N-glycosylation site in BxlB^wt^, previous studies have also demonstrated the critical importance of a single N-glycosylation site on folding and enzymatic activity. In *P. pastoris* BglS, for instance, N224 is important for enzyme activity and production, and N428 is essential for N-acetylglucosamine-6-sulfotransferase-1 activity [13, 40]. An increased number of positive correlations throughout the enzyme structure in BxlB^N1;5^ endorses the view that glycosylation may lead to a higher number of intramolecular contacts—mainly van der Waals packing contacts [41]. As a result, the protein movements acquire more of a rigid-body quality when compared to BxlB^non-glyc^ [42, 43]. However, the N1 and N5 N-glycosylation pattern also introduced negative correlations in the BxlB structure, suggesting an important change in the pattern of the essential dynamics of the enzyme that may underlie substrate binding, product release, and the observed differences in catalytic efficiency between BxlB^N1;5^ and BxlB^non-glyc^. Additional simulations, with different glycosylation patterns and different conditions, would be necessary to further analyze the role of *N*-glycans in BxlB.

### Changing BxlB^wt^ N-glycosylation context impairs protein folding and secretion

Our results show that the mispositioning of N-glycosylation sites impairs the production of recombinant enzymes in *A. nidulans*. To understand the impairment of BxlB^CC^ production, microscopy experiments to monitor enzyme secretion were performed showing that BxlB^CC^ mutations affect folding and secretion. The importance of balance in translation phases has been previously reported [44]. Thus, BxlB^CC^ misfolding likely triggered protein degradation, resulting in the secretion of only a small amount of a non-functional enzyme.

Proteins with difficulty in achieving the correct folding remain longer in the ER, which maintains strict quality control, either actively facilitating folding or degrading directing misfolded proteins [28, 45]. Quality control in *A. nidulans* is performed by the calnexin cycle. Calnexin recognizes *N*-glycans attached to proteins and assists disulfide bond formation, consequently facilitating correct folding and secretion [46, 47]. Here, confocal microscopy showed that BxlB^CC^ is retained in the intracellular fraction, suggesting that the mispositioning of N-glycosylation sites flagged the protein for degradation.

## Conclusions

The study of the effects of N-glycosylation patterns on fungal CAZymes contributes to understanding how *N*-glycan positioning affects protein structure, dynamics, stability, and function. Here, we demonstrate that N-glycosylation facilitates the correct folding of a GH3 β-xylosidase from *A. nidulans* by eliminating an intermediate state. Despite the relatively unaffected transcriptional levels, changing the N-glycosylation context hampered enzyme secretion. Secondary structures were preserved in BxlB glycomutants, but thermal stabilities were reduced. While BxlB^wt^ secretion and kinetic parameters were affected by N-glycosylation site mutations, the non-glycosylated enzyme (BxlB^non-glyc^) was secreted in a functional state. There is strong evidence that mispositioning BxlB N-glycosylation sites (BxlB^CC^) results in unfolded/misfolded non-functional enzyme. At an individual level, the N-glycosylation site N5 is essential for BxlB improved enzyme catalytic efficiency, but requires additional complementation by N1 and/or N7 sites. Moreover, BxlB glycomutants demonstrate the complexity of the N-glycosylation effect, positively and/or negatively affecting the folding process, secretion, and kinetic parameters. N-glycosylation engineering is a promising tool for enhancing target enzyme secretion, activity, and thermal stability.

## Materials and methods

### Strains, plasmids and media

The reference strain *A. nidulans* A773 (pyrG89;wA3;pyroA4) was purchased from the Fungal Genetics Stock Center (FGSC). *A. nidulans* A773 and recombinant strains were regularly maintained in minimal media (MM): 1% glucose (m/v), pH 6.5 [11, 48]. Plasmids were propagated in *E. coli* DH5α maintained in Luria–Bertani (LB) medium.

### Site-directed mutagenesis of GH3 β-xylosidase

The *bxlB*^wt^ gene was amplified from *A. nidulans* A773 genomic DNA by PCR using specific primers (Additional file 1: Table S6). The gene was cloned into the pEXPYR shuttle vector using the NotI and XbaI sites, as previously described [11].

The following amino acid substitutions in the BxlB^wt^ sequence were designed and genes synthesized by GenOne (Rio de Janeiro, Brazil): BxlB^N1;5;7^ (N340Q, N408Q, N419Q, N621Q), BxlB^non-glyc^ (N63Q, N340Q, N408Q, N419Q, N458Q, N621Q, N760Q), and BxlB^CC^ (N63Q, A123T, Q163 N, N340Q, Q391 N, N408Q, N419Q, L450T, N458Q, N621Q, N760Q). Despite the point mutation of some amino acids in the BxlB^CC^ to potential O-glycosylation sites, none of them were predicted as glycosylated. The other six mutants, BxlB^N1^ (N340Q, N408Q, N419Q, N458Q, N621Q, N760Q), BxlB^N5^ (N63Q, N340Q, N408Q, N419Q, N621Q, N760Q), BxlB^N7^ (N63Q, N340Q, N408Q, N419Q, N458Q, N621Q), BxlB^N1;5^ (N340Q, N408Q, N419Q, N621Q, N760Q), BxlB^N1;7^ (N340Q, N408Q, N419Q, N458Q N621Q), and BxlB^N5;7^ (N63Q, N340Q, N408Q, N419Q, N621Q) were designed using the Q5^®^ Site-Directed Mutagenesis Kit (New England Biolabs).

### Gene cloning and *A. nidulans* transformation

Genes cloned into the pEXPYR plasmid were transformed into calcium competent *E. coli* cells by heat shock, and confirmation was carried out using colony PCR. Positive colonies were cultivated overnight and then plasmids were extracted and used for fungal transformation.

Fungal transformation was carried out as previously described, with modifications [49]. Conidia from *A. nidulans* A773 were inoculated on YG medium (20 g·L^−1^ glucose, 5 g·L^−1^ yeast extract, 1 × trace elements, 1 mg·L^−1^ pyridoxine, 2.5 mg·L^−1^ uracil/uridine) and were incubated at 30 °C and 130 rpm for 13 h. Protoplasts were prepared by hydrolysis of the fungal cell wall with 125 mg lysozyme from chicken egg white (L7651, Sigma) and 1.02 g Vinotaste Pro (Novozymes) for 2 h. Approximately, 10 µg of recombinant DNA was mixed with the protoplast solution and 25% PEG (w/v). Protoplast recovery was performed on MM plates supplemented with 1.2 M sorbitol and pyridoxine, incubated at 37 °C for 3 days.

### LC–MS/MS validation of N-glycosylated sites

Purified N-glycomutants were incubated with 10 units of endoglycosidase-H (New England Biolabs) at 37 °C during 24 h for deglycosylation under denaturing conditions. Samples were then prepared for LC–MS/MS analysis as previously described [4]. To increase coverage and expand the number of peptide sequences, protein mutants were digested in gel with either 1 µg trypsin (Promega) at 37 °C overnight or 1 µg GluC (promega) at 37 °C overnight, and both simultaneously. The resulting peptides were concentrated by SpeedVac and 1 µL of each sample was injected for identification. Peptide loads were separated in an ACQUITY UPLC HSS T3 nanoACQUITY Column (100 Å, 1.8 µm, 75 µm X 150 mm, Waters Corporation) in one dimension across three steps of acetonitrile (7%, 40%, 85%) over 36 min at a flow rate of 500 nL/min directly into a Synapt G2-Si mass spectrometer (Waters Corp., Milford, USA). MS/MS analyses were performed by nano-electrospray ionization in positive ion mode with a NanoLock Spray (Waters Corporation) ionization source and collected in DIA mode. The lock mass channel was sampled every 30 s. The spectrometer was calibrated with an MS/MS spectrum of [Glu1]-fibrinopeptide B human (Glu-Fib) solution (100 fmol/µL) delivered through the reference sprayer of the NanoLock Spray source.

The N-glycosylation sites were validated by N-acetylglucosamine detection on asparagine residues by adding N-acetylglucosamine (*N*) as a variable modification (*N* + 203.0794) to the identification step performed by ProteinLynx Global Server (version 3.0.2; Waters Corp.). A manually created databank was used for identification with the peptide sequences of all expected protein variants. The FDR was set to 4% and was calculated using a reverse databank created on-the-fly. Relevant sequence coverage was validated within PLGS software (Additional file 6: Table S7).

### Structure based models and computational analysis

Structure Based Models (SBM) [50] were employed to evaluate the folding mechanisms and stability of non-glycosylated (BxlB^non-glyc^) and wild-type (BxlB^wt^) enzymes. In this model, the enzyme is represented by C_α_-beads and the N-glycosylations by beads in the center of mass of the saccharide rings and attached to amino acid (see details in SI). Although simple, C_α_-SBM is a suitable choice since they accurately describe the folding mechanisms for several proteins [51]. The native structure (or functional tridimensional form) was constructed using Swiss Model [52], based on *A. nidulans* β-xylosidase XlnD PDB 6Q7I (45.5% identity and 62.4% similarity).

The contact map of C_α_-SBM was defined by CSU-algorithm [51] and *N*-glycans were not considered. The simulations were carried out using the molecular dynamics software developed and tested by Whitford et al. [53]. Free energy (F) profiles and constant volume specific heat values were calculated using the weighted histogram analysis method (WHAM) [54] through simulations at various temperatures. The number of native contacts formed during the simulations (*Q*) was employed as a reaction coordinate to describe folding mechanisms. When *Q* is normalized by the total number of native contacts possible, values close to 1 indicate the *ensemble* of native conformations (nativeness).

The evaluation of how mutations interfere in the folding pathways was done by calculating *ϕ*-values, using the following equation [51, 55]:

$$\phi^{i} = \frac{{\Delta F_{\text{TS}}^{i} - \Delta F_{U}^{i} }}{{\Delta F_{N}^{i} - \Delta F_{U}^{i} }},$$

where Δ*F* is the free energy variation calculated by perturbation theory, i.e., evaluating the free energy difference of contacts in the presence and absence of residue *i.* These differences are related to the transition state (Δ*F*_TS_), native state (Δ*F*_N_) and unfolded state (Δ*F*_U_). *ϕ*-values close to 1 suggest poor mutation choices, since they can destabilize the transition state or folded state, affecting the folding pathway or folding rate. In some cases, the mutation of these residues can strongly interfere with the folding process, not allowing the protein to reach its functional state. Values close to 0 suggest that mutations of these residues may not interfere in the folding process, making them good candidates for mutations, especially for protein engineering using protein-surface residues. Furthermore, the *ϕ*-value calculations help to understand folding patterns, since they show contacts formed during the transition state for each residue.

### Molecular dynamics simulations

Molecular dynamics simulations were performed on the BxlB in both its N1;5 and non-glycosylated forms using the NAMD package [56] with CHARMM36 [57] force field for proteins and carbohydrates, and TIP3P water model [58]. The BxlB structure was modeled using the I-TASSER server [59], with the PDB structure 6Q7I [20] (45.5% identity and 62.4% similarity to BxlB) as a template and further refined with the GalaxyWEB server [60]. The hydrogen atoms of the enzyme were added according to the protonation state predicted by the H++ server [61, 62] at pH 5.5. The following residues were considered protonated (i.e., Asp/Glu residues electrically neutral and His residues positively charged): His67, His88, His144, Asp148, His175, His275, Asp288, His299, His300, Glu333, Glu489, Glu490, Glu510, Asp530, Glu582, Glu605, His616, and Asp649. The BxlB^N1;5^ glycosylated structure was modeled using the GLYCAM server [63], by covalently linking a Man_5_GlcNAc_2_ moiety to the Asn63 and Asn458 residues. Both BxlB^N1;5^ and BxlB^non-glyc^ structures were modeled with a xylobiose molecule in the active site, which was obtained from the PDB structure 5AE6 [to be published]. The simulation boxes contained, respectively to BxlB^N1;5^ and BxlB^non-glyc^, 31,178 and 29,621 water molecules, 119 and 115 sodium ions, and 88 and 84 chloride ions. Chloride and sodium ions were added to render the system electrically neutral at a salt concentration 0.15 mol L^−1^. The simulations were performed using periodic boundary conditions and the particle mesh Ewald algorithm [64] with a 12 Å cutoff and a smoothing function starting at 10 Å. Bonds involving hydrogen atoms were constrained at their equilibrium values and the simulation time step was 2.0 fs. All simulations were performed at a constant temperature of 310 K and a constant pressure of 1 bar, using the Langevin thermostat and pressure control. After initial relaxation and thermalization, both glycosylated and non-glycosylated systems underwent a production run of 400 ns each. Three additional runs 50 ns-long were carried out with both systems to check for unbinding events.

### Biochemical characterization of β-xylosidase and glycomutants

#### Enzymatic activity and protein determination

Fresh conidia from *A. nidulans* A773 and recombinant strains (10^7^–10^8^ per ml) were inoculated for 36 h at 37 °C in MM pH 6.5 supplemented with 2% (w/v) maltose. After growth, the mycelial mass was separated by filtration and the crude filtrate was centrifuged (10,000×*g*, 20 min 4 °C); mycelia were then washed and the intracellular content was extracted by maceration with liquid nitrogen, which was suspended in RIPA buffer and centrifuged (10,000×*g*, 20 min 4 °C). Protein concentration in the crude (non-purified) extracellular filtrate, as well as in the intracellular extract, was quantified by the Bradford method [65] and BCA method [66], respectively. About 20 µg of total protein from the supernatant was run in 10% SDS-PAGE [67]. Standard β-xylosidase activity was determined in 0.05 M ammonium acetate buffer pH 5.0 at 50 °C using 5 mM ρNP-X as a substrate. The reaction was stopped with 1 M sodium bicarbonate and the released *ρ*-nitrophenolate (ρNP) was determined at 400 nm. One unit (U) of β-xylosidase activity was defined as the amount of enzyme releasing 1 µmol of ρNP per minute under the assay conditions.

#### Protein purification

All enzymes were purified by two chromatography steps using the HiPrep™ DEAE FF 16/10 column (GE Healthcare), followed by a HiLoad™ 16/600 Superdex™ 200 pg column (GE Healthcare), as previously described [14]. Purification was evaluated by SDS-PAGE and protein concentration was determined by reading absorbance at 280 nm, using the molar extinction coefficient [31] calculated from the amino acid composition (http://web.expasy.org/protparam/) [68].

#### Structural characterization by circular dichroism (CD) spectroscopy

CD analysis was carried out using a JASCO 815 spectropolarimeter (JASCO Inc., Tokyo, Japan) equipped with a Peltier temperature control unit and a 0.1 cm path length cuvette, as previously described [69]. Data from 260 to 190 nm were collected at 100 nm/min scanning speed, 1 nm spectral bandwidth, and 0.5 s response time. Melting temperatures were evaluated by spectral measurement from 20 to 100 °C.

#### Effect of temperature and pH on enzyme activity

To determine the optimal temperatures of all recombinant proteins, enzyme activity was assayed as described above from 35 to 70 °C in 0.05 M ammonium acetate buffer, pH 5.0. Optimal pH was calculated by ranging the pH of a 0.1 M glycine–citrate–phosphate buffer from 3.0 to 12.5 at 50 °C, using ρNP-X as the substrate.

#### Kinetic parameters

Maximum velocity (*V*_max_), Michaelis–Menten constant (*K*_m_), the catalytic constant (*k*_cat_), and catalytic efficiency (*k*_cat_/*K*_m_) were determined for each of the mutants using different ρNP-X concentrations (1–20 mM). These analyses were performed at optimal conditions, 0.05 M ammonium acetate buffer, pH 5.0, and 60 °C.

### RNA extraction, transcript analysis by qPCR (quantitative real-time PCR), and primer design

*Aspergillus nidulans* total RNA was extracted by first grinding mycelia frozen in liquid nitrogen with a mortar and pestle, then collecting with a Direct-Zol RNA Miniprep kit (Zymo Research), according to the manufacturer’s instructions. Total RNA (DNA-free) was assayed for reverse transcription using the Maxima First Strand cDNA Synthesis Kit for RT-qPCR, with dsDNase (Thermo Fisher Scientific). cDNA samples were diluted, and each qPCR reaction containing cDNA (100 ng), SYBR Green (Life Technologies), forward and reverse primers (Additional file 1: Table S6), and nuclease-free water was carried out using the ViiA™ 7 real-time PCR system (Life Technologies). All PCR reactions were performed in biological triplicate. Gene expression levels were determined using the ΔΔCt method with β-tubulin (*tubC*) as the reference gene.

### Confocal microscopy

Microscopic analyses were carried out by confocal microscopy based on Fischer-Parton et al. with modifications [70]. Mycelium was obtained after cultivation of *A. nidulans* strains for 24 h and 48 h, at 37 °C and 200 rpm in MM. The images were obtained by an LSM 510 Axiovert 200 M (Carl Zeiss) confocal inverted microscope, fitted with an argon laser. Laser parameters were, respectively to cyan and red fluorescence, 365 and 590 nm excitation and 440 and 617 nm fluorescence emission, using the *A. nidulans* A773 strain to calibrate autofluorescence. All samples were spread over a microscope slide, *in natura*, then analyzed immediately after cultivation.

### Western blot

WB analysis was carried out as described by Nutzmann et al. [71]. Total protein (60 μg) was separated by SDS-PAGE and then transferred to PVDF membrane using a wet blotting system (Bio-Rad). The membrane was blocked with 5% BSA, incubated overnight with primary antibody (BxlB) and then gently shaken at room temperature following the addition of immunoglobulin G anti-rabbit secondary antibody labeled with peroxidase. Protein detection was carried out using the Clarity Western ECL Substrate chemiluminescence detection kit (Bio-Rad), as described by the manufacturer.

## Supplementary information

**Additional file 1.** Supplementary Tables and Figures.

**Additional file 2.** **Video S1**. BxlBwt production analysis by confocal microscopy. The polyclonal BxlB antibody was identified with a red-fluorescence antibody.

**Additional file 3.** **Video S2**. BxlBwt production analysis by confocal microscopy. The polyclonal BxlB antibody was identified with a red-fluorescence antibody.

**Additional file 4. Video S3**. BxlBcc production analysis by confocal microscopy. The polyclonal BxlB antibody was identified with a red-fluorescence antibody.

**Additional file 5.** **Video S4**. BxlBcc production analysis by confocal microscopy. The polyclonal BxlB antibody was identified with a red-fluorescence antibody.

**Additional file 6. Table S7**. LC-MS/MS analysis of BxlB glycomutants N-glycosylation sites.

## Data Availability

The datasets supporting the conclusions of this article are included in the article and its additional files.

## Abbreviations

GHs: glycoside hydrolases
CAZymes: carbohydrate-active enzymes
GH3: glycoside hydrolase family 3
ρNP-X: *ρ*-nitrophenyl β-d-xylopyranoside
ASA: accessible surface area
SP: signal peptide
CD: circular dichroism
MD: molecular dynamics
WB: western blot
FGSC: Fungal Genetics Stock Center
MM: minimal medium
LB: Luria–Bertani
CSU: contact of structural units
ρNP: *ρ*-nitrophenolate
PBS: phosphate-buffered saline
SBM: structure-based models
RMSF: root mean-square fluctuations
