# Supplementary material for: Redesigning N-glycosylation sites in a GH3 β-xylosidase improves the enzymatic efficiency
Source: Biotechnol Biofuels. 2019 Nov 14;12:269. doi: 10.1186/s13068-019-1609-2 (PMC6854716; doi:10.1186/s13068-019-1609-2)
Supplement: Supplementary file 1 — Additional file 1. Supplementary Tables and Figures. [file 13068_2019_1609_MOESM1_ESM.docx]

**Supporting data**

**Table S1. Overview of BxlB^wt^ putative N-glycosylated sites.**

| BxlB^wt^ (model based on PDB 6Q7I) | | | | | |
| --- | --- | --- | --- | --- | --- |
| **Asn number** | **Sequence** | **Asn position** | **ASA** | **N-glycosylation potential^1^** | **Experimental data**  **(LC-MS/MS)** |
| **1** | **KIN*NT** | **63** | **86.8 +** | **0.5549** | **Glycosylated** |
| **2** | **ISN*ET** | **340** | **54.8 +** | **0.6808** | **Glycosylated** |
| **3** | **KAN*GT** | **408** | **69.9 +** | **0.6643** | **Glycosylated** |
| **4** | **FIN*FT** | **419** | **60.4 +** | **0.3883** | **Glycosylated** |
| **5** | **EVN*ST** | **458** | **79.3 +** | **0.6399** | **Glycosylated** |
| **6** | **TFN*VS** | **621** | **71.0 +** | **0.4632** | **Glycosylated** |
| **7** | **SGN*DS** | **760** | **NC** | **0.4782** | **Glycosylated** |
| **BxlB^CC^ 1** | **AFN*DT** | **121** | **33.0 +** | **0.6883** | **ND** |
| **BxlB^CC^ 2** | **RGN*ET** | **166** | **NC** | **0.3211** | **ND** |
| **BxlB^CC^ 3** | **AIN*GT** | **391** | **NC** | **0.6974** | **ND** |
| **BxlB^CC^ 4** | **GYN*VT** | **448** | **73.6 +** | **0.6919** | **ND** |

ASA: Accessible Surface Area.

NC: not calculated.

ND: not determine.

^1^ Predicted by NetNGlyc 1.0 Server.

**Table S2. BxlB^CC^ peptides detected by LC-MS/MS.**

| Query | Observed | Mr (expt) | Mr (calc) | Delta | Score | Unique | Peptide |
| --- | --- | --- | --- | --- | --- | --- | --- |
| 1 | 378,6812 | 755,3479 | 755,349 | -0.0011 | 39 |  | RFEFDAKV |
| 2 | 444,2444 | 886,4742 | 886,476 | -0.0018 | 40 |  | KGDVQVLEKF |
| 3 | 480,271 | 958,5275 | 958,5488 | -0.0213 | 52 |  | RLPITQYPKS |
| 4 | 503,3022 | 1004,59 | 1004,602 | -0.0121 | 51 |  | RYLLQTVLRE |
| 5 | 544,7969 | 1087,579 | 1087,587 | -0.0081 | 68 |  | RVAEIISTEARA |
| 6 | 550,3059 | 1098,597 | 1098,607 | -0.0100 | 49 |  | KNIDWTLPLKA |
| 7 | 571,8564 | 1141,698 | 1141,707 | -0.0087 | 38 |  | KTVAIQGTVLLKN |
| 8 | 595,3351 | 1188,656 | 1188,66 | -0.0046 | 69 |  | KSLVSALTLEEKI |
| 9 | 622,8507 | 1243,687 | 1243,688 | -0.0015 | 48 |  | RRVAEIISTEARA |
| 10 | 626,811 | 1251,607 | 1251,625 | -0.0173 | 69 |  | KAFGPYDAATLARG |
| 11 | 634,3194 | 1266,624 | 1266,639 | -0.0147 | 12 |  | KHIPTMIEAAERL |
| 12 | 690,8664 | 1379,718 | 1379,72 | -0.0015 | 74 |  | KKAFGPYDAATLARG |
| 13 | 506,2389 | 1515,695 | 1515,691 | 0.0034 | 17 | U | KINNTGHEAAGSSRL |
| 14 | 528,2335 | 1581,679 | 1581,684 | -0.0055 | 26 |  | RGQETPGEDPLHCSRY |
| 15 | 808,4366 | 1614,859 | 1614,858 | 0.0009 | 15 | U | RQGLISQETLDAALTRL |
| 16 | 851,9121 | 1701,81 | 1701,811 | -0.0014 | 113 |  | KHLAAYDLEEWGGVSRF |
| 17 | 877,4689 | 1752,923 | 1752,934 | -0.0106 | 58 |  | KVSAVDLLEYYLPPFKT |
| 18 | 902,9621 | 1803,91 | 1803,921 | -0.0118 | 60 | U | RQGLISNETLDAALTRL |
| 19 | 910,4368 | 1818,859 | 1818,864 | -0.0045 | 50 | U | KGDVQVLEKFPLSGNDSD |
| 20 | 910,9232 | 1819,832 | 1819,848 | -0.0157 | 116 |  | RSLGWDDVATSEAEELAKT |
| 21 | 723,3626 | 2167,066 | 2167,085 | -0.0190 | 48 |  | RLGLPAYNWWNEALHGVAEKH |
| 22 | 756,3895 | 2266,147 | 2266,163 | -0.0166 | 59 |  | RLYTSLVQLGYFDPAEGQPLRS |
| 23 | 1234,5342 | 2467,054 | 2467,063 | -0.0093 | 87 |  | KSYVDEVPMTDMNLQPGTDNPGRT |

**Table S3. Circular dichroism analysis of BxlB glycomutants.**

|  | BxlB^wt^ | BxlB^N1;5;7^ | BxlB^non-glyc^ | BxlB^N1^ | BxlB^N5^ | BxlB^N7^ | BxlB^N1;5^ | BxlB^N1;7^ | BxlB^N5;7^ |
| --- | --- | --- | --- | --- | --- | --- | --- | --- | --- |
| **Tm** | 63.77 | 61.58 | 59.48 | 59.23 | 56.17 | 57.86 | 61.10 | 56.38 | 55.12 |
| **SD** | 1.21 | 0.644 | 0.427 | 0.677 | 0.805 | 0.823 | 2.07 | 0.462 | 0.547 |
| **Secondary structure** | α helix: 63.58% | α helix: 63.58% | α helix: 63.7% | α helix: 63.58% | α helix: 63.58% | helix: 63.58% | α helix: 63.58% | α helix: 63.58% | α helix: 63.58% |
|  | β strand: 12.55% | β strand: 12.54% | β strand: 12.59% | β strand: 12.59% | β strand: 12.55% | β strand: 12.55% | β strand: 12.54% | β strand: 12.55% | β strand: 12.55% |

**Table S4. Transition temperature based on C_v_ (T_c_) and folding temperature (T_f_) from simulations**.

| Mutant | C_v_ peak  (T in reduced units) | Folding temperature*  (T in reduced units) |
| --- | --- | --- |
| BxlB^wt^ | 1.144 ± 0.7 | 1.150 ± 0.02 |
| BxlB^non-glyc^ | 1.134 ± 0.7 | 1.132 ± 0.02 |
| BxlB^N1;5;7^ | 1.150 ± 0.7 | 1.152 ± 0.02 |
| BxlB^N5;7^ | 1.134 ± 0.7 | 1.134 ± 0.02 |
| BxlB^N1^ | 1.134 ± 0.7 | 1.136 ± 0.02 |

* From Free energy profiles – **Figure S6.**

**Table S5. β-xylosidases characterized using ρNP-X (according to MycoClap*).**

| Entry Name | Species | Host (for recombinant expression) | Specific Activity | pH | Temp  (°C) | Genbank Protein ID | Uniprot ID | Literature PMID |
| --- | --- | --- | --- | --- | --- | --- | --- | --- |
| XYL3A_TRIRE | *Trichoderma reesei* | *Saccharomyces cerevisiae* | active | - | - | CAA93248 | Q92458 | 8837440 |
| XYL3D_ASPNG | *Aspergillus niger* | native | 60.2 U/mg | - | - | CAB06417, CAB59162, CAK37179, CAW52627 | O00089 | 9128738 |
| XYL3A_EMENI | *Emericella nidulans* | *Aspergillus nidulans G191* | active | 5.0 | 50 | CAA73902 | - | 9546179 |
| XYL3A_ASPOR | *Aspergillus oryzae* | native | 76 U/mg | 4.0 | 60 | BAA28267 | - | 9872754 |
| XYL3A_ASPAW | *Aspergillus awamori* | native | active | - | - | BAE19756 | Q4AEG8 | 16202538 |
| XYL3A_ASPJA | *Aspergillus japonicus* | native | 112 U/mg | 4.0 | 70 | BAG82824 | B6EY09 | 19000618 |
| XYL3A_AURPU | *Aureobasidium pullulans* | native | 288 U/mg | 3.5 | 70 | BAI82526 | D4AHT5 | 20547381 |
| XYL3A_TRIRE | *Trichoderma reesei* | native | 28 U/mg | 4.0 | 60 | - | - | DOI: 10.1007/BF00268208 |
| XYL3A_ASPAW | *Aspergillus awamori* | native | 20 U/mg | 4.0 | 70 | - | - | DOI: 10.1271/bbb.61.2010 |
| XYL3A_EMENI | *Emericella nidulans* | native | 107 U/mg | - | - | - | - | http://onlinelibrary.wiley.com/doi/10.1111/j.1574-6968.1996.tb08003.x/pdf |
| XYL3A_ASPJA | *Aspergillus japonicus* | *Pichia pastoris GS115* | 19 U/mg | - | - | - | - |  |

* MycoClap site: https://mycoclap.fungalgenomics.ca/mycoCLAP/

**Table S6. Oligonucleotides used in this study.**

| Primer name | Sequence |
| --- | --- |
| BxlB^wt^ Fwd | 5’- TATA**GCGGCCGC**TACCCGGACTGCACAA -3’ |
| BxlB^wt^ Rev | 5’- TATA**TCTAGA**TAATCACTGTCGTTACCTGACA -3’ |
| RT BxlB^wt^ Fwd | 5’- ACCATGATCGAGGCAGCAG -3’ |
| RT BxlB^wt^ Rev | 5’- GGGCATCATCGAATCCGTCT -3’ |
| N1 Fwd | 5’- AGAGAAAATCAACAACACGGGCCACG -3’ |
| N1 Rev | 5’- TCGAGTGTCAGGGCAGAG -3’ |
| N5 Fwd | 5’- GACAGAGGTGAATTCGACAAGCACAGACG -3’ |
| N5 Rev | 5’- CCTGGCGCGGTGAGAACG -3’ |
| N7 Fwd | 5’- GTTGTCAGGTAACGACAGTGATTATC -3’ |
| N7 Rev | 5’- GGAAACTTTTCAAGCACC -3’ |

*Not*I and *Xba*I restriction sites in bold.

**SM1 - C_α_-Structure Based Model construction**

Structure Based Models (SBM), also known as Gö-like models, are interesting approaches to evaluate protein folding mechanisms. The construction is based in the tridimensional functional form or crystallographic (native state), so it is not a structural predictive method based on primary or secondary sequences, *i.e*., the native state must be known.

The C_α_-SBM is the simplest construction, being the amino acid residue represented by its C_α_ position. The force field is based on the defined native structure (Γ_0_) relative to the current conformation (Γ), and given by:

$V\left( \Gamma,\Gamma_{0} \right)= \sum_{bonds} \frac{1}{2}\varepsilon_{r}\left( r- r_{0} \right)^{2}+\sum_{angles} \frac{1}{2}\varepsilon_{\theta}\left( \theta- \theta_{0} \right)^{2}+\sum_{dihedrals} \left\{ \varepsilon_{\phi}\left[ 1-cos\left( \theta-\theta_{0} \right) \right]+\frac{1}{2}\varepsilon_{\phi}\left[ 1-cos(3\left( \theta-\theta_{0} \right)) \right] \right\}+ \sum_{\left| i-j \right|>3 contacts} \varepsilon_{LJ}\left[ {5\left( \frac{\sigma_{ij}}{r_{ij}} \right)}^{12}-6\left( \frac{\sigma_{ij}}{r_{ij}} \right)^{10} \right]$ + $\sum_{non-contacts} \varepsilon_{repulsive}\left( \frac{\sigma_{ij}}{r_{ij}} \right)^{12}$

The first three terms are the mathematical modeling of covalent bonds, geometrical restrain of organic models and information about the secondary structure (chirality). *r* is the distance between two bonded beads, *θ* is the angle formed between two adjacent bonds and *ϕ* is the angle formed between two planes of four bounded consecutively beads. *ε_r_ = 100* *kT, ε_θ_ = 20* *kT*, *ε_ϕ_ = 1* *kT*, were *k* is the Boltzmann constant and *T* the temperature. The *r_0_*, *θ_0_* and *ϕ_0_* are the reference values obtained from the native structure. The fourth term is the Lennard Jones potential where contacts are defined based on the work of Sobolev and coworkers (CSU contact map algorithm) for the atoms *i* and *j* separated in the primary sequence by *| i - j | > 3*. *σ_ij_* is the distance in the native structure and *r*_ij_ the distance between them. If a pair of atoms is not defined as *in contact* by CSU contact map, it is considered a non-contact (steric restraint) and the *σ_ij_* is set as 4Å and ε*_repulsive_*=*1 kT*. Saccharides included by glycosylation are not considered in the contact map. The total number of native contacts normalized is defined as *Q* (nativeness, close to 1 folded and close to 0 unfolded).

**
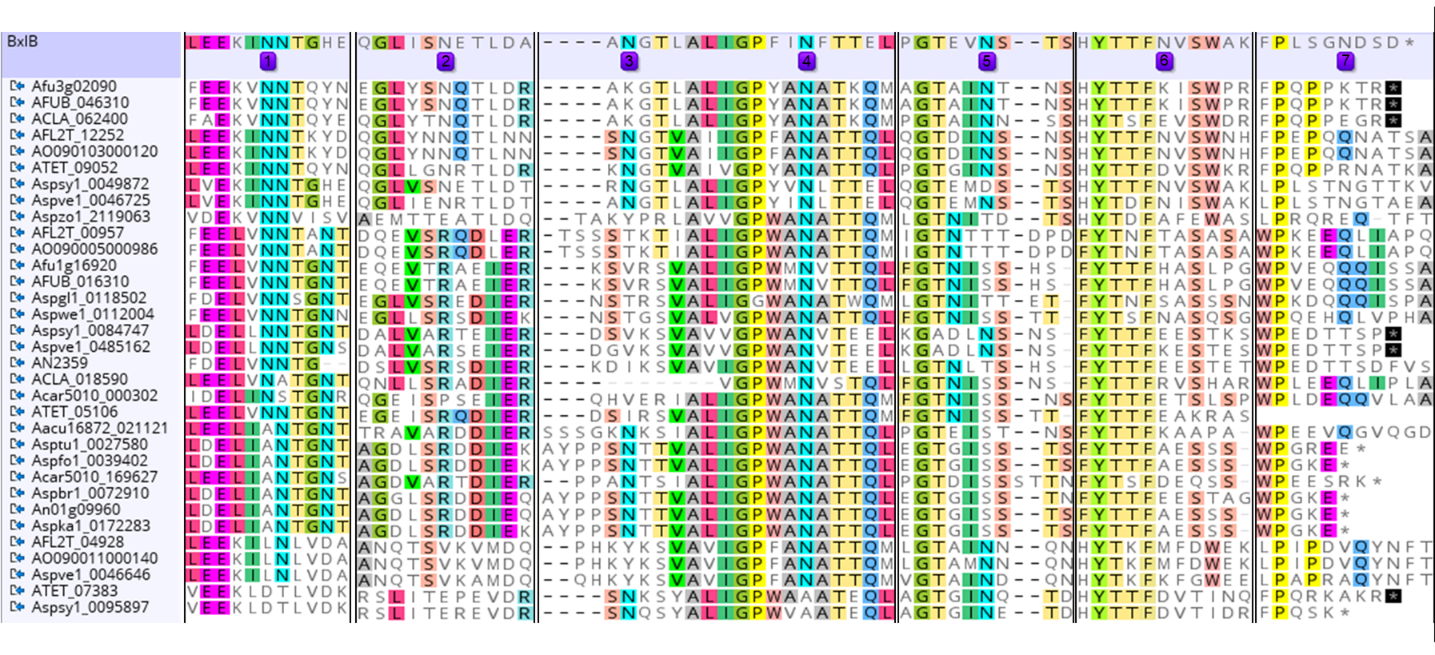
**


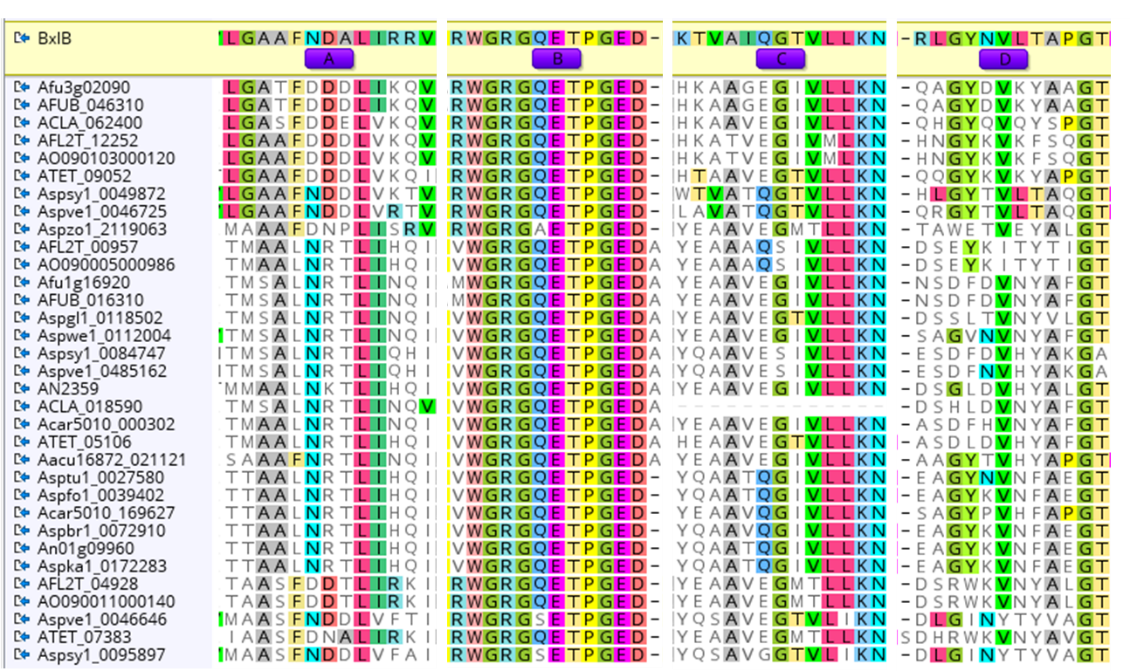


**Figure S1. Analysis of N-glycosylation sites conservation by alignment of 33 BxlB^wt^ homologous sequences.** Alignment performed using the ClustalW algorithm. Homologous sequences (E-value < 10^-40^) obtained from the Aspergillus Genome Database (AspGD). BxlB^wt^ N-glycosylation sites are indicated by positions 1 to 7 and four new BxlB^CC^ N-glycosylation sites are indicated by position A to D. Aacu16872 *Aspergillus aculeatus* ATCC16872; Acar5010 *Aspergillus carbonarius* ITEM 5010; ACLA *Aspergillus clavatus* NRRL 1; AFL2T *Aspergillus flavus* NRRL 3357; Afu1g *Aspergillus fumigatus* Af293; AFUB *Aspergillus fumigatus* A1163; An01g *Aspergillus niger* CBS 513.88; AN *Aspergillus nidulans* FGSC A4; AO0900 *Aspergillus oryzae* RIB40; Aspbr1 *Aspergillus brasiliensis* CBS 101740; Aspfo1 *Aspergillus acidus* CBS 106.47; Aspgl1 *Aspergillus glaucus* CBS 516.65; Aspka1 *Aspergillus kawachii*; Aspsy1 *Aspergillus sydowii*; Asptu1 *Aspergillus tubingensis* CBS 134.48; Aspve1 *Aspergillus versicolor*; Aspwe1 *Aspergillus wentii* DTO 134E9; Aspzo1 *Aspergillus zonatus*; ATET *Aspergillus terreus* NIH2624.

**Figure S2. Analysis of BxlB glycomutants intracellular activity by homologous expression in *A. nidulans*.** The β-xylosidase activity was measured using the ρNP-X assay. The reaction was carried out for 15 min at 50 °C and pH 5.0. A773: reference *A. nidulans* strain. *ANOVA, *p*<0.05. * *p* ≤ 0.05; ** *p* ≤ 0.002; *** *p* ≤ 0.0002 and **** *p* ≤ 0.0001

**Figure S3. BxlB glycomutants real-time PCR analysis.** Data analyzed by the ΔΔCt method using the *tubC* gene as endogenous gene and *A. nidulans* A773 as control strain. Gene induction was performed on 2% maltose for 36 h. ANOVA analysis showed no significant difference.

**
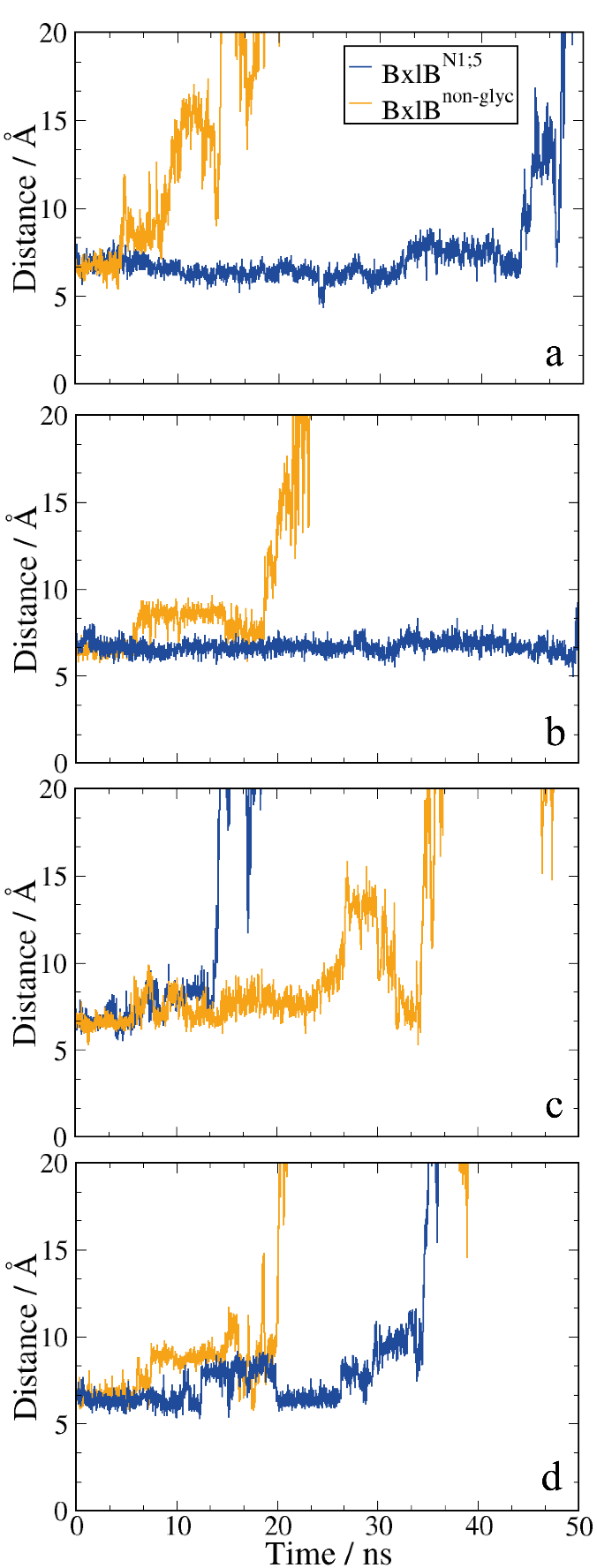
.**

**Figure S4. Distances between the glycoside bond oxygen and the OD2 atom from Asp288 over four independent 50 ns-long simulations.** a) First 50 ns of the 400 ns-long simulation, when the xylobiose ligand stayed approximately four times longer in the binding pocket. b,c,d) 50 ns-long replicates that started from the same initial conditions as “a” and support the hypotheses of a hindered substrate release in the presence of N-glycans at N1 and N5.

**Figure S5. BxlB glycomutants circular dichroism spectra.** Glycomutants structural analysis was conducted by CD using a JASCO J-815 spectropolarimeter.


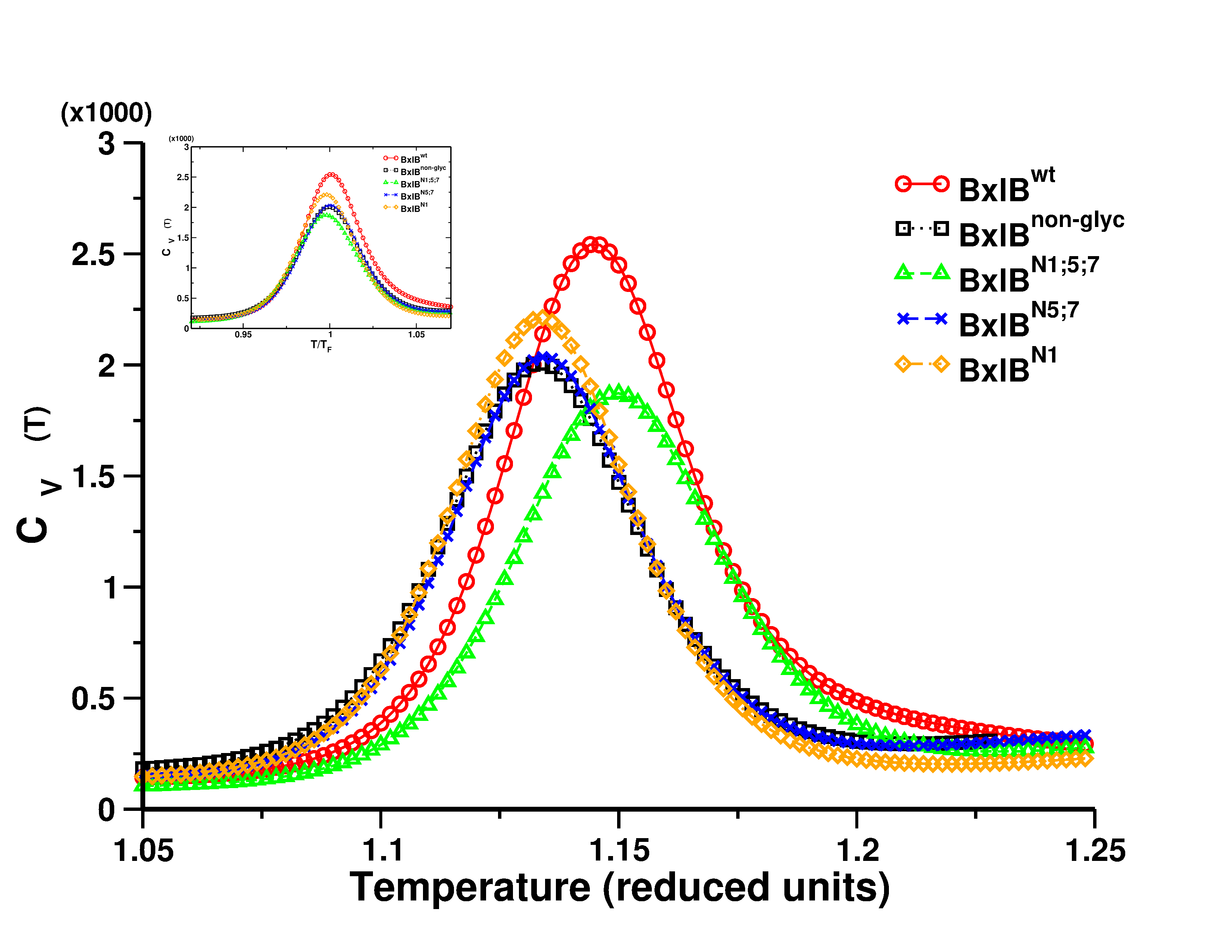


**Figure S6.** **Heat capacity at constant volume (C_v_) of BxlB^wt^, BxlB^non-glyc^, BxlB^N1;5;7^, BxlB^N5;7^ and BxlB^N1^**. The peaks identify the critical temperatures for the transition, revealing that the wild-type form (*T = 1.14 in reduced units*) and BxlB^N1;5;7^ are thermically more stable than the non-glycosylated form (*T = 1.13 in reduced units*) based on the melting temperature (T_m_). The result corroborates with the experimental assays (**Table S3** and **Figure S5**) and are detailed in **Table S4**. The inset shows the curves with the temperature normalizes by the critical temperature, indicating a slight less cooperative folding for the wild-type form.


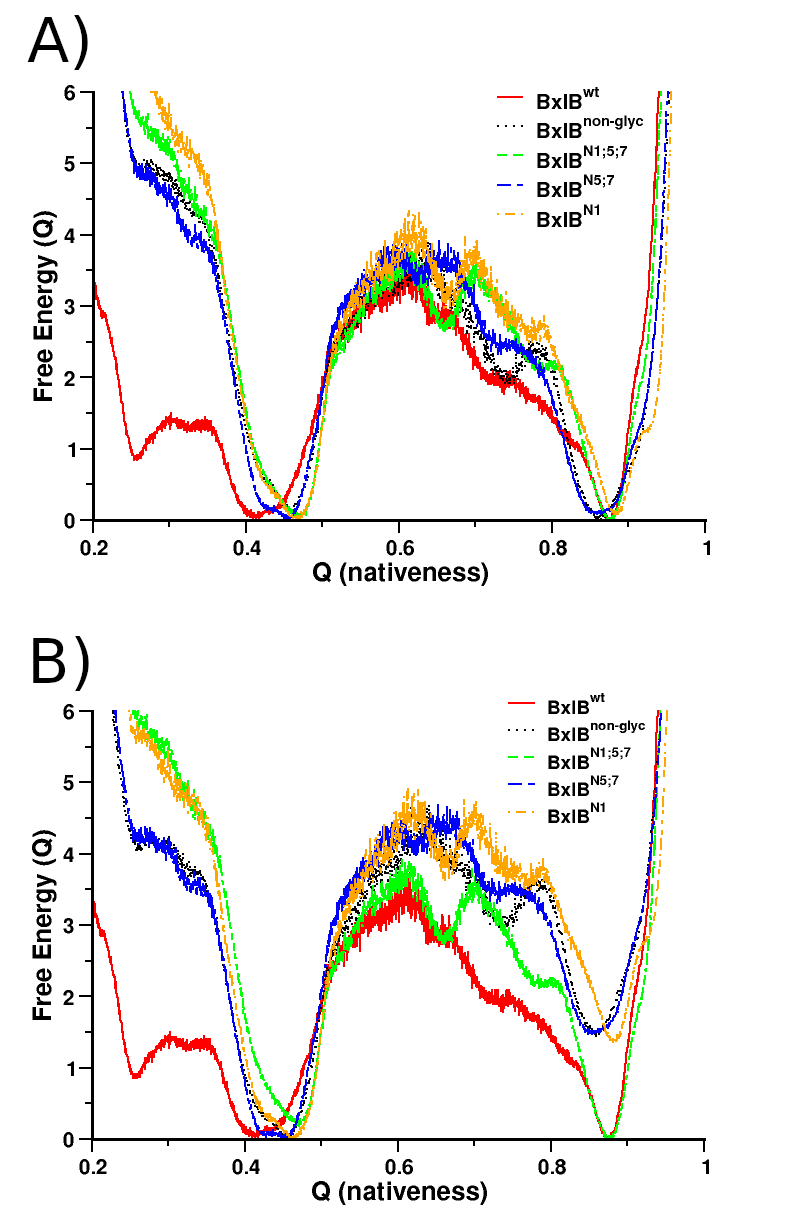


**Figure S7.** **Free energy analysis of BxlB^wt^, BxlB^non-glyc^, BxlB^N1;5;7^, BxlB^N5;7^ and BxlB^N1^. A)** The free energy profile was obtained against the protein nativeness (number of native contacts, *Q*) using Weighted Histogram Method (WHAM). The curves are presented in the Folding Temperature (*Tf*) of the enzyme indicated (see **Table S4**). The region in *x-axis* around *Q = 0.85* and *Q=0.45* indicates the folded and unfolded conformational *ensembles*, respectively. The peaks close to *Q = 0.60* define the transition state, where intermediated states were observed for all cases except BxlB^wt^. Thus, the BxlB^wt^ indicates that N-glycans facilitate folding process, since the enzyme do not remains trapped in the transition state. The wild-type form still has a lower barrier (*3.4 kT*) compared with the other models, highlighting the role of glycosylation. **B)** Free energy profile with the temperature fixed in T=1.15 in reduced units (*T_f_* of BxlB^wt^ model). The curves allow the comparison of folded and unfolded populations between all constructions computationally evaluated and support the hypothesis of higher thermal stability to BxlB^wt^ and BxlB^N1;5;7^.


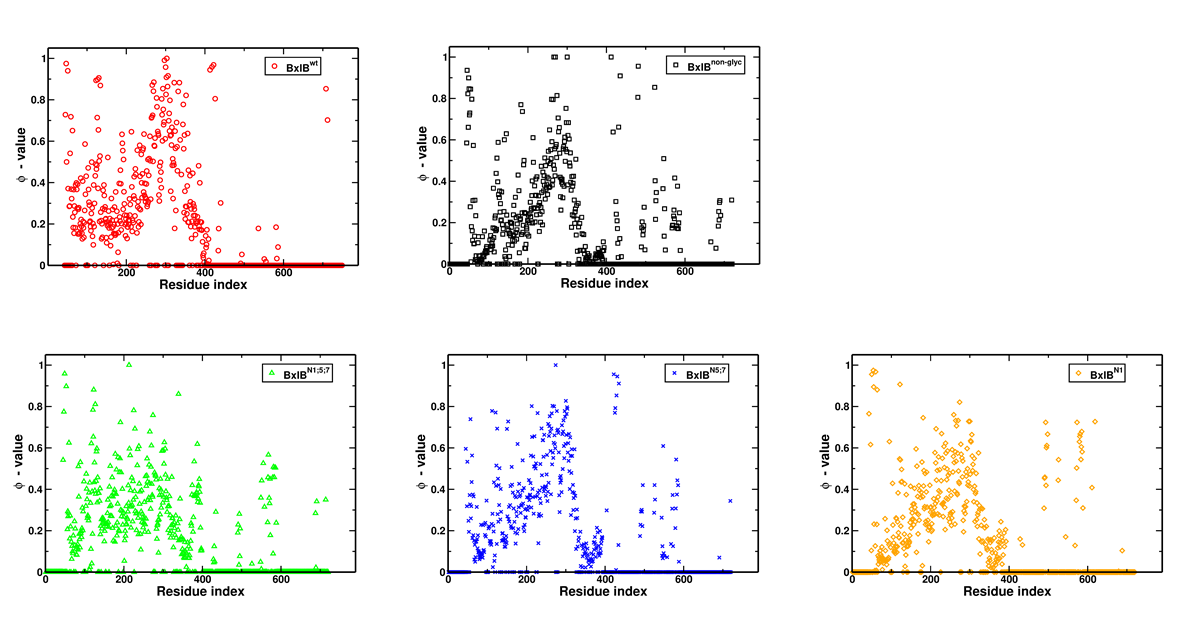


**Figure S8.** **Evaluation of the transition state of BxlB^wt^, BxlB^non-glyc^, BxlB^N1;5;7^, BxlB^N5;7^ and BxlB^N1^.** *ϕ-values* calculations evaluate the importance of each residues during the folding process in the transition state (TS). Values close to 1 indicates essential residues that should not be mutated, otherwise they may interfere in the mechanisms to reach the native form, being values close to 0 referent to residues that not participate of interactions during the TS. The calculations were performed using perturbation theory and shows a more coordinated folding process to BxlB^wt^, since the N-terminal protein portion (residues from 1 to 400) is formed previously to later the C-terminal portion (residues with index 400 or higher) to be formed. Consequently, the folding route is understood as more specific and favorable to BxlB^wt^ when compared to the other cases, based in the lower free energy barrier (**Figure S6**). In the opposite situation, the BxlB^non-glyc^ reveals a folding process where in the TS residues of the C-terminal portion are involved in the folding process, resulting in the occurrence of intermediate states. Thus, the wild-type glycosylations set contribute entropically to the protein folding process by the conformational space restriction in TS, not allowing the C-terminal portion be formed prematurely. The analysis of BxlB^N1;5;7^, BxlB^N5;7^ and BxlB^N1^ also provide insights about the how the reduction of the BxlB^wt^ N-glycosylations change the mechanisms of folding in the TS.

**
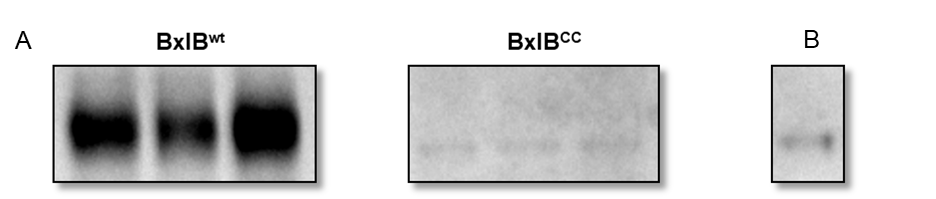
**

**Figure S9. WB analysis of BxlB glycomutants.** (A) *A. nidulans* intracellular fraction were analyzed, using an anti-BxlB polyclonal antibody, in triplicate using 100 μg of total proteins and 0.5 μg of purified BxlB^wt^ as a positive control (B).


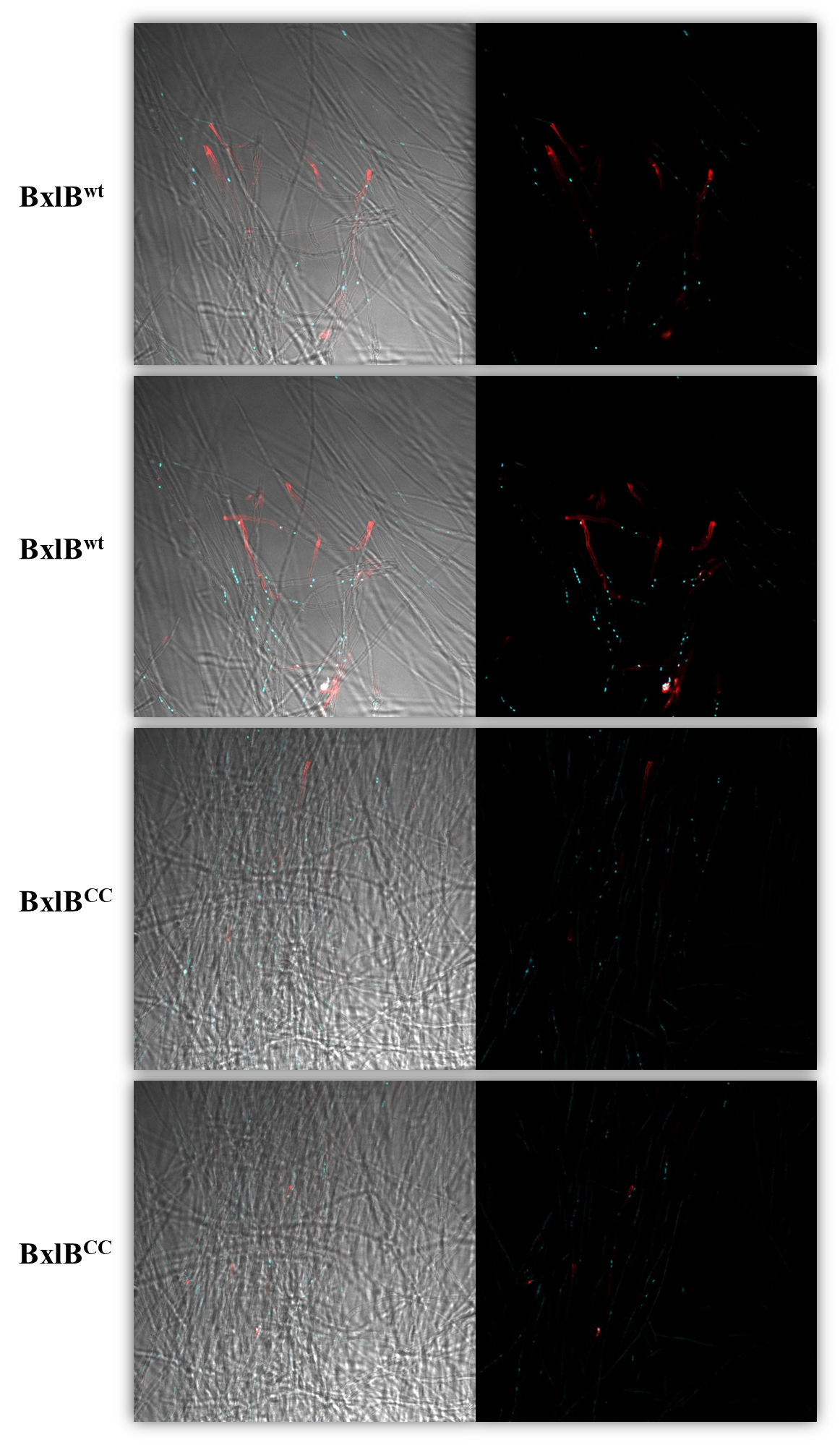


**Figure S10. Monitoring of BxlB mutants by immunohistochemistry, using confocal microscopy.** Monitoring intracellular BxlB glycomutants began after 24 h induction in 2% maltose. Z-stack were captured to each glycomutant, however just two stacks were represented here. Pictures were captured at the same stack with white light and lasers (left) and just lasers (right). All images were captured using the same parameters. Red: BxlB glycovariants. Cyan: nucleus. Watch the Z-stack videos in the supplementary data.
